# 14-3-3 Proteins in Glutamatergic Synapses

**DOI:** 10.1155/2018/8407609

**Published:** 2018-04-23

**Authors:** Jiajing Zhang, Yi Zhou

**Affiliations:** Department of Biomedical Sciences, Florida State University College of Medicine, Tallahassee, FL 32306, USA

## Abstract

The 14-3-3 proteins are a family of proteins that are highly expressed in the brain and particularly enriched at synapses. Evidence accumulated in the last two decades has implicated 14-3-3 proteins as an important regulator of synaptic transmission and plasticity. Here, we will review previous and more recent research that has helped us understand the roles of 14-3-3 proteins at glutamatergic synapses. A key challenge for the future is to delineate the 14-3-3-dependent molecular pathways involved in regulating synaptic functions.

## 1. Introduction

14-3-3 refers to a family of homologous proteins that consist of seven genetic loci or isoforms (*β*, *γ*, *ε*, *η*, *σ*, *ζ*, and *τ*) in vertebrates. The name 14-3-3 was given based on the fraction number and migration position on DEAE-cellulose chromatography and subsequent starch-gel electrophoresis during its initial biochemical purification process [1]. 14-3-3 proteins exist as homo- or heterodimers, in which each 14-3-3 monomer shares a similar helical structure and forms a conserved concave amphipathic groove that binds to target proteins via specific phosphoserine/phosphothreonine-containing motifs [2–7]. Through protein-protein interactions, 14-3-3 functions by altering the conformation, stability, subcellular localization, or activity of its binding partners. To date, 14-3-3 proteins have been shown to interact with hundreds of proteins and are implicated in the regulation of a multitude of cellular processes [8, 9].

14-3-3 proteins are highly expressed in the brain, comprising ~1% of its total soluble proteins. Thus, it comes to no surprise that 14-3-3 proteins are involved in a variety of neuronal processes, such as neurite outgrowth, neural differentiation, migration and survival, ion channel regulation, receptor trafficking, and neurotransmitter release [10–12]. In addition, 14-3-3 proteins are genetically linked to several neurological disorders, including neurodegenerative diseases (e.g., Parkinson's, Alzheimer's, and Creutzfeldt-Jakob diseases), neurodevelopmental diseases (e.g., Lissencephaly), and neuropsychiatric disorders (e.g., schizophrenia and bipolar disorder) [13–15], thus making them a potential therapeutic target [16, 17]. In recent years, a number of small molecule 14-3-3 modulators have been discovered that could be used to either stabilize or inhibit 14-3-3 protein-protein interactions [18, 19]. However, as 14-3-3 proteins are involved in diverse cellular processes, it is highly desirable to further characterize and develop compounds that have enhanced isoform specificity as well as can selectively modulate the 14-3-3 interaction with a critical target in a particular pathway.

14-3-3 proteins are generally found in the cytoplasmic compartment of eukaryotic cells. In mature neurons, however, certain 14-3-3 isoforms are particularly enriched at synapses, suggesting their potential involvement in synaptic transmissions [20, 21]. Indeed, evidence accumulated in the last two decades reveals that 14-3-3 is an important modulator of synaptic neurotransmissions and plasticity. In this review, we will discuss the functional role of 14-3-3 proteins in the regulation of glutamatergic synapses.

## 2. Functions of 14-3-3 at the Presynaptic Site

Early evidence that 14-3-3 might regulate synaptic transmission and plasticity came from genetic and functional studies of the fruit fly *Drosophila*. The gene *leonardo* encodes 14-3-3*ζ*, one of the two *Drosophila* 14-3-3 homologs that is abundantly and preferentially expressed in mushroom body neurons. Mutant *leo* alleles with reduced 14-3-3*ζ* proteins exhibit significant deficits in olfactory learning and memory, suggesting a functional role of 14-3-3 in these processes [22]. A subsequent study further determined that the 14-3-3*ζ* protein progressively accumulates to the synaptic boutons during maturation of the neuromuscular junction (NMJ), where it colocalizes with the synaptic vesicles containing the neurotransmitter glutamate [23]. Based on electrophysiological analyses, *Leonardo* mutants show impaired presynaptic functions at NMJ, including reduced endogenous excitatory junctional currents (EJCs), impaired transmission fidelity, and loss of long-term augmentation and posttetanic potentiation (PTP). The evoked transmission deficit in *leo* is more severe under lower external Ca^2+^ concentration, suggesting a possible defect in Ca^2+^-dependent presynaptic transmission in the absence of 14-3-3*ζ* proteins.

Following those studies in *Drosophila* NMJs, the involvement of 14-3-3 proteins in the presynaptic site of glutamatergic synapses was further investigated in the vertebrate nervous system. One potential mechanism is thought to be mediated by 14-3-3 binding to RIM1*α*, an active zone protein that is essential for presynaptic short- and long-term plasticity [24, 25]. Early biochemical studies have provided the first evidence that 14-3-3 binds to RIM1*α* through its N terminal domain, raising the possibility that 14-3-3 regulates neurotransmitter release and synaptic plasticity through the regulation of RIM1*α* [26]. A later study further confirmed this protein-protein interaction and identified that PKA phosphorylation of serine-413 at RIM1*α* (pSer413) is critical for 14-3-3 binding [27]. Moreover, electrophysiological assays in cultured cerebellar neurons suggested that recruitment of 14-3-3 to RIM1*α* at pSer413 is required for a presynaptic form of long-term potentiation (LTP) at granule cell and Purkinje cell synapses in the mouse cerebellum [27–29]. However, apparently contradictory evidence came from later efforts to examine the involvement of 14-3-3 and RIM1*α* interaction in presynaptic long-term plasticity using *in vivo* animal models. In one of these studies, a line of knock-in mice was generated to substitute RIM1*α* serine-413 with alanine (S413A), thereby abolishing RIM1*α* phosphorylation at S413 and 14-3-3 binding. Surprisingly, electrophysiological examination of the RIM1*α* S413A knock-in mice failed to detect a significant defect in presynaptic LTP, either at parallel fiber or mossy fiber synapses [30]. In agreement with this finding, an acute *in vivo* rescue experiment showed that deficits of mossy fiber LTP in RIM1*α*^−/−^ mice can be rescued by expression of the phosphorylation site-deficient mutant of RIM1*α* (S413A) [31]. Thus, it remains unclear whether 14-3-3 binding to S413 phosphorylated RIM1*α* plays a significant role in the regulation of presynaptic long-term plasticity.

**Figure 1:**
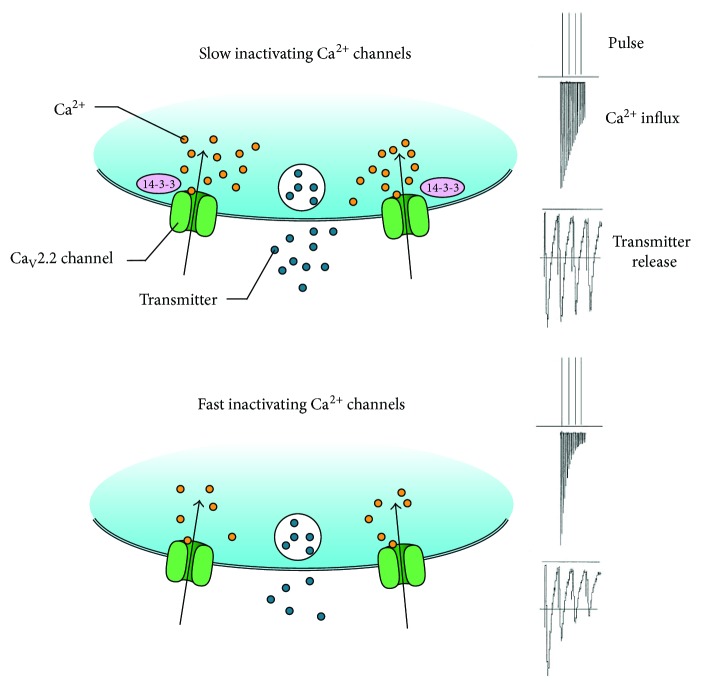
14-3-3 regulates presynaptic short-term plasticity by modulating Ca_V_2.2 channel properties. 14-3-3 binding reduces cumulative inactivation of Ca_V_2.2 channels and sustains Ca^2+^ influx and neurotransmitter release (a). Inhibition of 14-3-3 accelerates Ca_V_2.2 channel inactivation and enhances short-term synaptic depression (b).

A better-understood action of 14-3-3 at the presynaptic site is its role as the modulator of ion channels [32, 33], which include voltage-gated calcium (Ca^2+^) channels that play a central role in neurotransmitter release by mediating Ca^2+^ influx at nerve terminals [34]. In particular, Ca_V_2.2 channels undergo cumulative inactivation after a brief, repetitive depolarization, thus markedly impacting the fidelity of synaptic transmission and short-term synaptic plasticity [35, 36]. 14-3-3 modulates inactivation properties of Ca_V_2.2 channels through its direct binding to the channel pore-forming *α*_1B_ subunit. In cultured rat hippocampal neurons, inhibition of 14-3-3 proteins in presynaptic neurons augments short-term depression, likely through promoting the closed-state inactivation of Ca_V_2.2 channels (Figure 1) [37]. As 14-3-3 binding can be regulated by specific phosphorylation of the *α*_1B_ subunit, this regulatory protein complex may provide a potential mechanism for phosphorylation-dependent regulation of short-term synaptic plasticity.

## 3. Functions of 14-3-3 at the Postsynaptic Site

The role of 14-3-3 at the postsynaptic site emerged more recently from the studies of various 14-3-3 mouse models. One of them, the 14-3-3 functional knockout (FKO) mice, was generated by transgenic expression of difopein (dimeric fourteen-three-three peptide inhibitor) that antagonizes the binding of 14-3-3 proteins to their endogenous partners in an isoform-independent manner, thereby disrupting 14-3-3 functions [38–41]. Transgene expression is driven by the neuronal-specific Thy-1 promoter which produces variable expression patterns in the brains of different founder lines, making it possible to assess the behavioral and synaptic alterations associated with expression of the 14-3-3 inhibitor in certain brain regions [42, 43]. Inhibition of 14-3-3 proteins in the hippocampus impairs associative learning and memory behaviors and suppresses long-term potentiation (LTP) at hippocampal CA3-CA1 synapses of the 14-3-3 FKO mice [41]. Through comparative analyses of two different founder lines with distinct transgene expression patterns in the subregions of the hippocampus, it was further determined that postsynaptic inhibition of 14-3-3 proteins may contribute to the impairments in LTP and cognitive behaviors. These observations thus revealed a postsynaptic function for 14-3-3 proteins in regulating long-term synaptic plasticity in mouse hippocampus.

**Figure 2:**
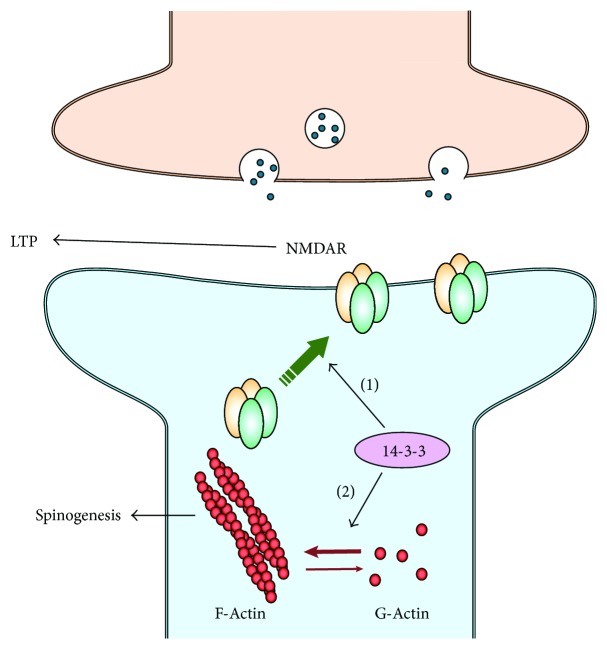
14-3-3 regulates NMDA receptors and actin dynamics at postsynaptic sites. (1) 14-3-3 proteins facilitate targeting of NMDARs to the postsynaptic density, thereby regulating long-term potentiation; (2) 14-3-3 proteins might promote spinogenesis by facilitating F-actin formation.

What might be the molecular targets of 14-3-3 proteins at the postsynaptic site of hippocampal synapses? In the 14-3-3 FKO mice, there is a significant reduction of the NMDA receptor-mediated synaptic currents in CA1 pyramidal neurons which express the 14-3-3 inhibitor. Consistently, the level of NMDA receptors (NMDARs), particularly GluN1 and GluN2A subunits, is selectively reduced in the postsynaptic density (PSD) fraction of 14-3-3 FKO mice that exhibit deficits in cognitive behaviors and hippocampal LTP [41]. Considering the critical role that NMDARs play in mediating LTP at hippocampal CA3-CA1 synapses [44], 14-3-3 proteins likely exert their effects on postsynaptic sites through the regulation of NMDA receptors, either directly or indirectly (Figure 2).

NMDARs are heterotetramers composed of two obligatory GluN1 subunits and two regulatory subunits derived from GluN (GluN2A-2D) and GluN3 subunits [45, 46]. 14-3-3 is known to promote surface expression of NMDA receptors in cerebellar neurons through its interaction with PKB-phosphorylated GluN2C subunits [47]. A more recent study also showed that inhibiting endogenous 14-3-3 proteins using difopein greatly attenuate GluN2C surface expression in cultured hippocampal neurons [48]. However, it remains to be determined whether 14-3-3 proteins directly interact with other subunits of NMDAR and have similar effects on their surface expression. Alternatively, 14-3-3 might indirectly regulate the PSD level of NMDARs by modulating other critical steps of NMDAR synaptic trafficking, such as dendritic transport and synaptic localization [45, 49]. Therefore, further studies are needed to better understand the exact mechanism underlying 14-3-3 proteins' regulation of NMDA receptors. Interestingly, the synaptic level of certain 14-3-3 isoforms is reduced in GluN1 knockdown mice, but not by subchronic administration of an NMDAR antagonist in wild-type mice [50]. It raises a possibility that a reciprocal regulation between 14-3-3 and NMDARs may take place at the postsynaptic site.

14-3-3 proteins also modulate other glutamate receptors at the postsynaptic membrane. For example, 14-3-3 interacts with GluK2a, a subunit of the kainate receptor (KAR) that mediates postsynaptic transmission, synaptic plasticity, and neuronal excitability [51]. 14-3-3 binding slows desensitization kinetics of GluK2a-containing KARs. In 14-3-3 FKO mice, expression of the 14-3-3 inhibitor in CA3 neurons leads to a faster decay of KAR-EPSCs at hippocampal mossy fiber-CA3 synapses [52]. This study provides another potential mechanism by which 14-3-3 proteins regulate synaptic functions at the postsynaptic site.

In addition to modulating the level and biophysical properties of postsynaptic glutamate receptors, 14-3-3 functions by regulating synaptogenesis. In the 14-3-3 FKO mice, there is a reduction of both dendritic complexity and spine density in the cortical and hippocampal neurons where the 14-3-3 inhibitor is extensively expressed [53]. A similar reduction in dendritic spine density was observed in 14-3-3*ζ*-deficient mice in BALB/c background [54, 55]. On the contrary, overexpressing 14-3-3*ζ* in rat hippocampal neurons significantly increases spine density [56]. Collectively, studies on these animal models provide *in vivo* evidence for a significant role of 14-3-3 proteins in promoting the formation and maturation of dendritic spines.

While the molecular mechanism for 14-3-3 dependent regulation of synaptogenesis remains elusive, several *in vitro* studies have proposed 14-3-3 proteins as important regulators of cytoskeleton and actin dynamics, which are critical for controlling the shape, organization, and maintenance of dendritic spines in postsynaptic neurons [57, 58]. Earlier studies showed that 14-3-3*ζ* regulates actin dynamics through its direct interaction with phosphorylated cofilin (p-cofilin) [57]. Cofilin is a major actin depolymerizing factor. Reduction of p-cofilin enhances the activity of cofilin, promotes the turnover of actin filaments, and consequently destabilizes dendritic spines [55, 56]. Moreover, a different group identified cofilin and its regulatory kinase LIM-kinase 1 (LIMK1) as binding partners of 14-3-3*ζ* and suggested that interactions with the C-terminal region of 14-3-3*ζ* inhibit the binding of cofilin to F-actin [59]. However, a direct interaction between 14-3-3 and cofilin/p-cofilin was challenged by a later study, in which Sudnitsyna et al. demonstrated that 14-3-3 only weakly interacts with cofilin, and they suggested that 14-3-3 proteins most likely regulate actin dynamics through other regulatory kinases such as LIMK1 or slingshot 1 L phosphatase (SSH) [60]. In fact, 14-3-3*ζ* has been shown to directly bind with phosphorylated SSH and lower its ability to bind F-actin [58].

More recently, Toyo-oka et al. showed that 14-3-3*ε* and 14-3-3*ζ* bind to *δ*-catenin and potentially regulate actin dynamics through *δ*-catenin [11, 61]. Catenin activates the Rho family of GTPase that results in the phosphorylation and activation of LIMK1. Loss of 14-3-3 proteins results in stabilization of *δ*-catenin through the ubiquitin-proteasome system, thereby decreasing LIMK1 activity and reducing p-cofilin level. Therefore, it is possible that 14-3-3 proteins may promote F-actin formation and spinogenesis by interacting with multiple elements in the regulatory pathways of the actin polymerization/depolymerization cycles (Figure 2).

## 4. Conclusion

The glutamatergic synapses mediate the majority of excitatory neurotransmission in the mammalian brain. Regulation of the property and connectivity of glutamatergic synapses represents a major mechanism for activity-dependent modification of synaptic strength and is critical for higher brain functions. 14-3-3 proteins have emerged as one of the important modulators at these synapses. It is particularly interesting that 14-3-3 binding and function are generally regulated by phosphorylation, which is a well-established molecular process underlying synaptic plasticity. Thus, 14-3-3 can potentially integrate multiple signaling pathways and plays a significant role in dynamic modification of glutamatergic synapses. As demonstrated by recent animal models, 14-3-3 deficiencies in rodent brain often result in the onset of abnormal behaviors, which might correspond to symptoms of neurological disorders.

## Abbreviations

NMJ: Neuromuscular junction
EJCs: Excitatory junctional currents
EPSCs: Excitatory postsynaptic currents
PTP: Posttetanic potentiation
LTP: Long-term potentiation
difopein: Dimeric fourteen-three-three peptide inhibitor
FKO: Functional knockout
PSD: Postsynaptic density
LIMK: LIM kinases
SSH: Slingshot.
